# Vaccination as a Significant Factor Influencing the Psychoemotional State of Medical Students During the Sars-Cov-2 Pandemic: An International Aspect

**DOI:** 10.2174/1745-0179-v19-e230420-2022-49

**Published:** 2023-07-11

**Authors:** Maria V. Sankova, Vladimir N. Nikolenko, Tatiana M. Litvinova, Beatrice A. Volel, Marina V. Oganesyan, Andjela D. Vovkogon, Negoria A. Rizaeva, Sergey V. Sankov, Mikhail Y. Sinelnikov

**Affiliations:** 1 First Moscow State Medical University named after I.M.Sechenov, Sechenov University, st. Trubetskaya, 8, bld. 2, Moscow 119991, Russian Federation; 2 Lomonosov Moscow State University, Leninskie Gory 1, Moscow 119991, Russia; 3 Research Institute of Human Morphology, Moscow, Russia

**Keywords:** SARS-COV-2 pandemic, Medical students, SARS-COV-2 vaccination, Increasing collective immunity, Psycho-emotional state, Education quality

## Abstract

**Background:**

The rapid spread of SARS-COV-2, characterized by its severe course in the absence of effective specific treatment for this infection, may become a significant risk factor for psycho-emotional disorders' emergence during this pandemic. One of the vulnerable groups in the current situation are first-year medical students, whose problems associated with an unfavorable sanitary-epidemiological situation and an increased infection risk are compounded by the difficulties of adapting to specific professional environments. In this situation, along with strict adherence to nonspecific prevention methods, the mass student SARS-COV-2 vaccination acquires particular importance.

**Objective:**

To compare the attitudes of first-year medical students in Russia and Azerbaijan toward SARS-COV-2 immunization and to assess the vaccination impact on the student's psycho-emotional state during the SARS-COV-2 pandemic.

**Materials and Methods:**

The study involved 594 first-year students at the Moscow and Baku branches of Sechenov University. The Google Forms platform was used to conduct an anonymous sociological survey. To compare the psychoemotional state of vaccinated freshmen and non-vaccinated students, we used the State-Trait Anxiety Inventory, STAI, to assess reactive anxiety and the Beck Depression Inventory test − to diagnose depressive symptoms. The online survey was conducted during the fourth wave of coronavirus infection. WHO official sources were used to analyze the current epidemiological SARS-COV-2 situation during the study data provided by the Russian Federal Service on Customers’ Rights Protection and Human Well-Being Surveillance and JHU CSSE. Statistical analysis was carried out using RStudio.

**Results:**

The study results showed that vaccination coverage of first-year students at the Moscow branch of Sechenov University during the fourth wave of the SARS-COV-2 pandemic was 42,9±5,13%, at the Baku branch − 69,6±5,86%. The lack of reliable information about anticovid vaccines, indicated by a third of all respondents, may largely determine the motivated participation in the vaccination SARS-COV-2 campaign. The role of medical school in imparting knowledge about active SARS-COV-2 immunization to medical students was found to be insignificant. It was shown that the percentage of students willing to recommend SARS-COV-2 vaccination to the people around them and thereby contribute to increasing collective immunity level significantly depends on the percentage of students vaccinated. It was proved that vaccinated students were characterized by significantly greater psychological stability regardless of their study place.

**Conclusion:**

Vaccination is not only a good preventive measure against the infection spread but also a significant factor in stabilizing the psycho-emotional state of first-year students, which significantly affects the quality of their educational process and its effectiveness.

## 1 INTRODUCTION

The global expansion of the new coronavirus SARS-CoV-2 infection, characterized by lightning-quick lesions of large lung tissue volumes, progressive respiratory failure development and numerous extrapulmonary complications, was recognized as a pandemic by the World Health Organization in March 2020 [1-4]. The rapid SARS-COV-2 spread, new, more pathogenic strains emerging, and the restrictive measure introduction are accompanied by significant changes in millions of people's lifestyles and financial status [5-7]. This, combined with constant concern for loved ones, the negative information flood, and the lack of effective specific treatment for SARS-CoV-2, could be a significant risk factor for psycho-emotional disorders in the current pandemic [8-11]. One of the vulnerable groups in this situation are first-year medical students, whose problems related to the unfavorable sanitary-epidemiological situation and an increased infection risk are superimposed on the not-yet consolidated adaptation to the critical moment and difficulties associated with professional specifics [12-14]. It was shown that under prolonged negative factors, there is a violation of the adaptive capabilities and the emergence of anxiety-depressive disorders, which significantly impact the educational process effectiveness and the further professional career of future specialists [15, 16]. In this situation, along with strict adherence to nonspecific prevention methods, the mass student SARS-COV-2 vaccination acquires particular importance, which is a necessary safeguard against moderate and severe forms of this disease, deaths and post-infectious complications [17-19]. In Moscow, COVID-19 vaccination began on December 5, 2020, and in Baku, on January 18, 2021. Medical students were among the first in this campaign since they have a high infection risk [17].

### 1.1 Objective

To compare the attitudes of first-year medical students in Russia and Azerbaijan toward SARS-COV-2 immunization and to assess the vaccination impact on the student's psycho-emotional state during the SARS-COV-2 pandemic.

## 2 MATERIALS AND METHODS

### 2.1 Study Design and Participants

**Fig. (1):**
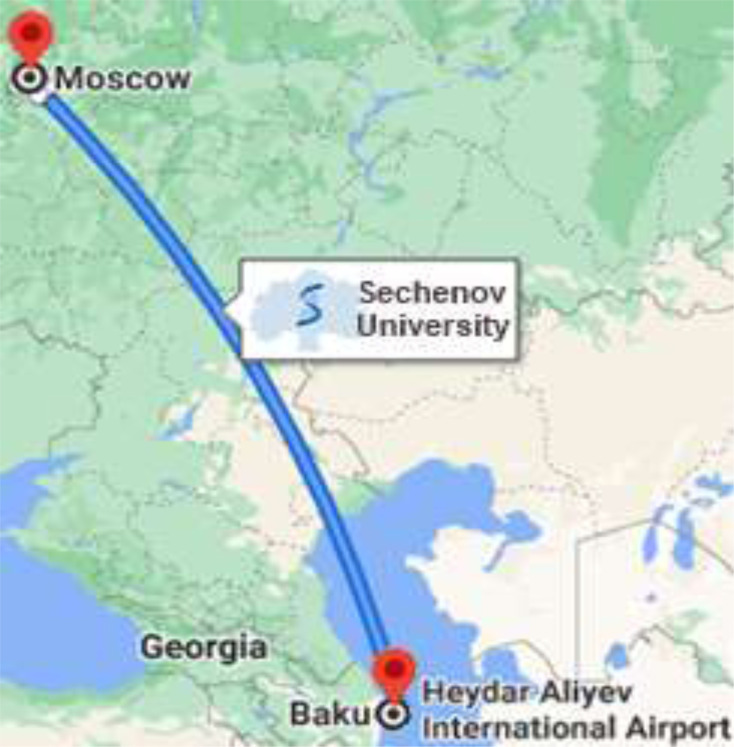
Moscow and Baku Sechenov University branches.

The cross-sectional online anonymous survey was carried out in the period of late October-early November 2021, during the fourth wave of coronavirus infection.). By this time, students could be vaccinated against COVID-19 for more than six months after administering the vaccine. The study involved 594 first-year medical students at the Moscow and Baku branches of Sechenov University (Table **1**), located in Russia and Azerbaijan, the regions with different epidemiological SARS-CoV-2 situations (Fig. **1**). The average age of the respondents was 17.89±0.61 years.

A Google Forms platform (Alphabet, USA), per STROBE guidelines, was used to conduct the anonymous sociological questionnaire, the reference to which was distributed *via* social networks and multifunctional applications. Using G*Power software statistical package (ChristianAlbrechts-Universität, Olshausenstr, Germany) [20] and based on a moderate effect size of 0.3, power 90%, and alpha<0.05, the minimum sample size needed for this study was calculated to equal 504 students. The formula used, and the minimum sample size calculations are shown below.


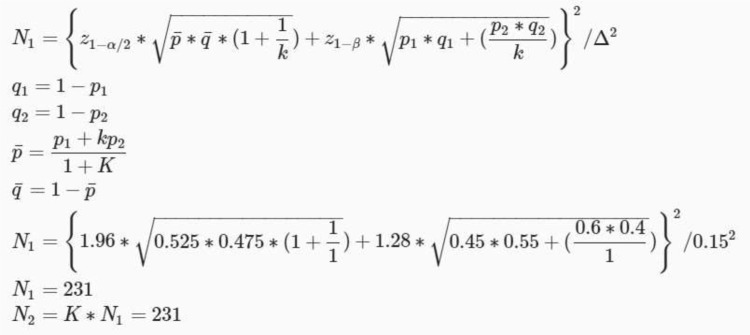


p1, p2 = proportion (incidence) of groups #1 and 2

Δ = |p2-p1| = absolute difference between two proportions

n1 = sample size for group #1

n2 = sample size for group #2

α = probability of type I error (usually 0.05)

β = probability of type II error (usually 0.2)

### 2.2 Epidemiological Situation Assessment

The up-to-date data provided by Russian Federal Service on Customers’ Rights Protection and Human Well-Being Surveillance and JHU CSSE, WHO official sources were used to analyze the current epidemiological SARS-COV-2 situation for SARS-COV-2 during the survey in Moscow and Baku, respectively. It was shown that Moscow compared to Baku, had a higher frequency in the number of new coronavirus infection incidence, mortality and lethality rates, which can lead to more pronounced stress reactions among metropolitan residents, including medical university students. The percentage of vaccinated adults in the Moscow region did not significantly differ from that in Azerbaijan at the time of the study (Table **2**).

### 2.3 Questionnaire Content

The original questionnaire was designed at Sechenov University. All questions were checked to be clear and easy to understand by three independent, experienced experts [21]. At first, students fill out personal information such as institutional affiliation, residence, age and sex. The main part of the questionnaire consists of four sections. The first contains questions about COVID-19 assessment, previous COVID-19 experience in relatives and themselves, attitudes towards non-specific SARS-CoV-2 preventive measures and their effectiveness evaluation. In the second section, student beliefs about COVID-19 vaccination were studied. When determining the effectiveness of vaccination and non-specific SARS-CoV-2 preventive measures, the student used a visual analog scale, in which 10 points corresponded to the effectiveness maximum and 0 points to its complete absence. The third section focuses on participant COVID-19 vaccine preference and vaccine information sources. Finally, we investigated COVID-19 vaccination coverage among first-year medical students, side effects after COVID-19 vaccination and predominant reasons for not vaccinating. In the last questions, respondents could choose several options. At last State-Trait Anxiety Inventory, **STAI** was used to assess reactive anxiety and the Beck Depression Inventory test to diagnose depressive symptoms [22, 23].

### 2.4 Ethical Considerations

The study was approved by the Local Ethics Committee of Sechenov University (Protocol No. dated) and the Helsinki Declaration norms. All students were recruited on a volunteer basis and gave informed consent before the study. Respondents understood the survey's purpose and were told how to complete the questionnaire. No reward was offered to participants.

### 2.5 Statistical Analysis

Statistical methods included processing the obtained data using RStudio software. The minimal subject number needed for this study was calculated using power analysis. The difference significance in the obtained results was analyzed using Student's t-test, Pearson’s χ2 and Fischer's exact test. The results were considered statistically significant at p <0.05. The results were counted twice by 2 independent researchers. The inter- and intragroup correlation agreement rates were more than 95%; for this reason, all results were considered the mean between 2 tries for 2 researchers.

## 3 RESULTS

### 3.1 Participant COVID-19 Experience

**Fig. (2):**
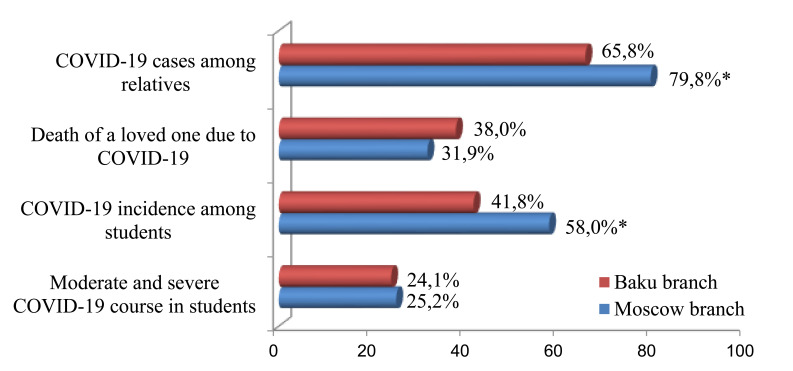
Participant COVID-19 experience, * - p<0.05.

An online survey showed that almost all first-year students of both the Moscow and Baku branches (91.6±2.88% and 94.9±2.81%, respectively) of Sechenov University considered COVID-19 a life-threatening disease and are concerned about the ongoing third-year SARS-COV-2 pandemic. Most first-year students have experienced this infection in their relatives and Moscow significantly more often than in Baku. And every third student, both in Moscow and Baku, has survived the death of a loved one (Fig. **2**).

Despite the strict observance of non-specific preventive measures by almost all first-year students in Moscow and Baku (95.0±2.26% and 98.7±1.44%, respectively), about half of them suffered from COVID-19, with a significantly higher percentage of those in Moscow than in Baku. Every fourth infected person from the Moscow and Baku branches suffered the disease in a moderate and severe form. Students living in the family were significantly more in Baku than in Moscow (87.3±4.24% and 60.5±2.59%, respectively, p<0.05).

### 3.2 First-year Student Beliefs About COVID-19 Vaccination

**Fig. (3):**
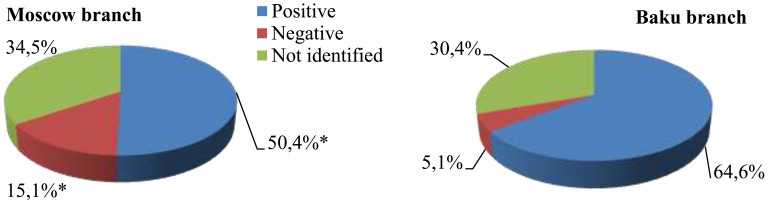
Attitudes of first-year students toward SARS-COV-2 vaccination, * - p<0.05.

It was shown that in both branches of Sechenov University, every third student doubted the efficacy and safety of the rapidly developed SARS-COV-2 vaccines. Only half of Moscow's first-year medical students have a positive attitude toward COVID-19 vaccination. A significantly higher percentage of respondents (almost two-thirds) decided to be vaccinated in Baku. A significantly higher percentage of young people with negative attitudes toward SARS-COV-2 vaccination was observed among Moscow students (Fig. **3**).

**Fig. (4):**
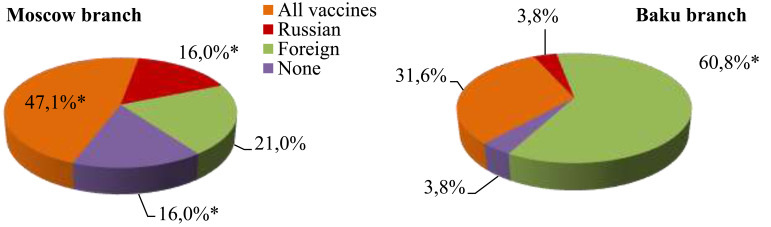
First-year student attitude toward SARS-COV-2 vaccines, * -p<0.05.

First-year students studying at the Moscow branch were significantly more likely to express the opinion that vaccines have guaranteed quality regardless of the origin country. Russian students preferred Russian-produced vaccines in more cases, while Azerbaijani students were more likely to trust foreign vaccines. It should be emphasized that Moscow first-year students are convinced that all immunobiological agents are ineffective in a greater percentage of cases. Every fifth Russian student would like to be vaccinated with a foreign-made vaccine (Fig. **4**).

**Fig. (5):**
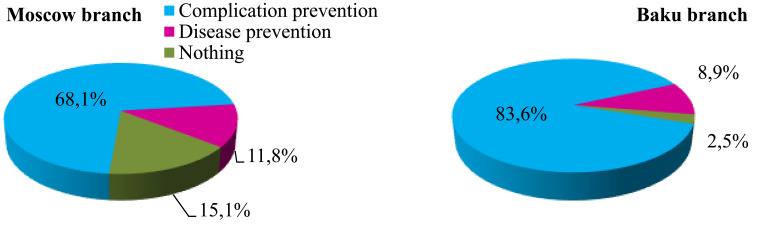
First-year student definition of the SARS-COV-2 vaccination role, * - p<0.05.

The significance of active immunization against SARS-COV-2 incidence in the assessment of both Russian and Azerbaijani first-year students did not differ from the effectiveness of non-specific preventive measures against this infection (Moscow 6.06±2.80 and 5.78±2.32% respectively; Baku 6.24±2.23% and 6.09±2.42% respectively). Thus, most first-year students in Russia and Azerbaijan believed that SARS-COV-2 vaccination guarantees protection against moderate and severe forms of this pathology, deaths and post-infection complications. Only one in ten respondents note that active immunization may prevent infection and the development of this disease. A greater percentage of first-year Russian students considered vaccination generally ineffective (Fig. **5**).

**Fig. (6):**
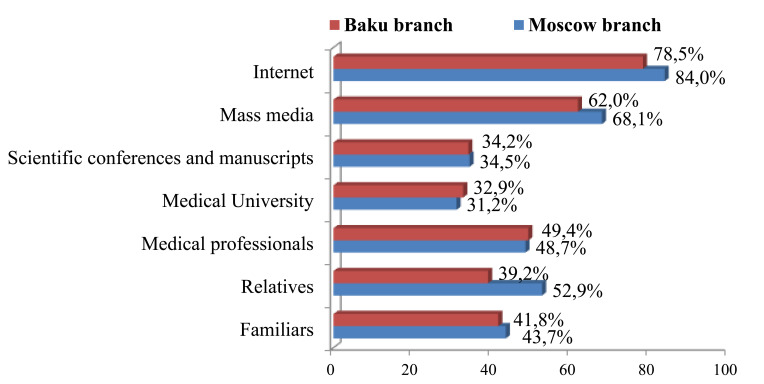
Sources of information on SARS-COV-2 vaccination in first-year students.

It was shown that first-year students' attitudes toward SARS-COV-2 vaccine prophylaxis are shaped mainly by information from the Internet and the mass media (Fig. **6**).

The position of half of the respondents was determined according to the information received from medical professionals. The least importance in gaining knowledge about COVID-19 preventive vaccines, their composition, action mechanisms and contraindications are noted for medical universities, scientific conferences and manuscripts, the role of which is underlined only by a third of students, that is comparable to the number of students whose information source are familiars and relatives. Lack of reliable information about COVID-19 vaccines, which could lead to a subsequent motivated decision to participate in the SARS-COV-2 vaccination campaign, was emphasized by a third of all first-year students regardless of their study place (32.7±2.48 and 32.8±3.05, respectively).

### 3.3 COVID-19 Vaccination among First-year Medical Students and Reasons for Not Vaccinating

**Fig. (7):**
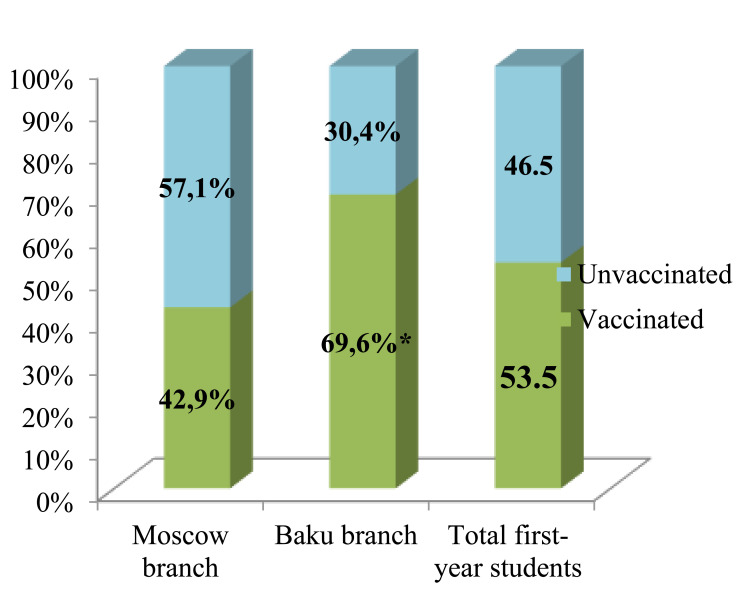
The proportion of vaccinated and unvaccinated first-year students, * - p<0.05.

The real COVID-19 vaccination rate among first-year medical students in autumn 2021 was 53.53±7.78%. The immunization coverage of students at the Baku branch was found to be significantly higher than the percentage of students who completed the full vaccination course in Moscow (Fig. **7**).

Most Russian first-year students for SARS-COV-2 vaccination chose the combined two-component vaccine “Gam-COVID-Vac” (“Sputnik V”) (72.5±4.63%) and a single-component vaccine Sputnik- Light (9.80±3.08%) developed at the N.F. Gamaleya Federal Research Center for Epidemiology & Microbiology based on adenovirus vectors. Every tenth Moscow student was vaccinated with Covivac (9.90±3.09%), an inactivated whole-virion vaccine. Peptide vaccines EpiVacCorona and EpiVacCorona H (State Research Center of Virology and Biotechnology VECTOR) were the least popular among Russian youth (7.80±2.78%) [24].

In Azerbaijan, the vaccine “Gam-COVID-Vak” (“Sputnik V”) was chosen by 41.7±3.20% of first-year medical students. The rest of the students (58.3±6.28%) used for immunization foreign anti-covid vaccines AstraZeneca of British-Swedish production based on adenovirus vector, BioNTech/Pfizer developed by the American company Pfizer and its German partner BioNTech based on matrix RNA, and CoronaVac of a Chinese biopharmaceutical company, representing inactivated whole-virion vaccine [19].

**Fig. (8):**
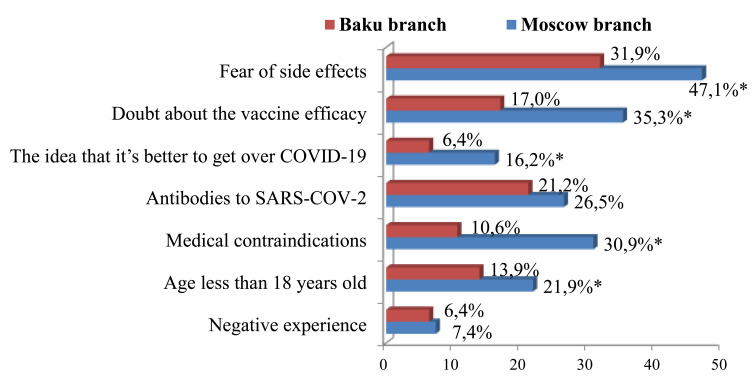
The main reasons for student refusal from SARS-COV-2 vaccination, * -p<0.05.

Among the dominant reasons for refusing SARS-COV-2 vaccination in first-year medical students at Moscow and Baku branches were the fear of side effects and post-vaccination complications, the lack of awareness about vaccines, and doubts about their effectiveness and safety (Fig. **8**).

Every fifth student from Russia, who had not reached adult age at the time of the study, could not be vaccinated due to the absence of a drug developed for this age group in Russia. It should be noted that unjustified refusal to vaccinate was more common among Russian students, who, in turn, were significantly less likely to recommend active SARS-COV-2 immunization to their friends, familiars and relatives than Azerbaijani students (58.8±5.13% and 77.2±5.34% respectively, p<0.05).

### 3.4 Psycho-emotional Status Of Sars-Cov-2 Vaccinated and Unvaccinated First-year Students

One of the main criteria for assessing student adaptive capabilities is the level of their reactive anxiety, which did not differ significantly in the first-year students at the Moscow and Baku branches (40.75±3.35 and 39.13±4.58 scores, respectively) and corresponded to a reaction of moderate severity, which was combined with signs of mild degree reactive depression (9.76±2.79 and 10.38±2.36 scores respectively). A comparison of students psychoemotional state depending on their behavioral attitude towards SARS-COV-2 vaccination revealed that the vaccinated students were characterized by significantly greater psychological stability.

Thus, unvaccinated students, both in Russia and Azerbaijan, were characterized by a high level of reactive anxiety (45.02±3.20 and 46.17±3.29 points, respectively), which led to additional stress on adaptive mechanisms and significantly destabilized the psychoemotional state of first-year students, contributing to the development of depressive disorders (11.85±1.94 and 12.08±2.06 scores, respectively), the level of which corresponded to the interval of mild depression indicators [22]. Vaccinated first-year students of the Moscow and Baku branches did not have depressive disorders (7.62±2.09 and 7.98±1.96 points, respectively) and had moderate anxiety (37.39±3.07 and 38.08±2.98 points, respectively).

## 4 DISCUSSION

This study found that almost all students, both Moscow and Baku branches, consider COVID-19 a life-threatening disease. Vaccination is well known to be the most reliable and effective method to prevent the spread of serious vaccine-preventable infections, including SARS-COV-2. The real COVID-19 vaccination rate among first-year medical students at Sechenov University in autumn 2021 was 53.53±7.78%, significantly lower than the target value for collective immunity, determined at 80% by the Russian Federal Service on Customers’ Rights Protection and Human Well-Being Surveillance. Similar results indicating an insufficient SARS-COV-2 vaccination rate among medical students were reported in several other studies, including our own [21, 25].

It was found that the COVID-19 vaccination rate was significantly higher in first-year medical students than in the adult population of both Moscow and Baku due to the greater awareness of medical students about COVID-19 severity and available treatment options emphasized in other studies [17, 21, 25]. Moscow had a lower vaccination rate than Baku. This may be because, among Moscow students, more people have already had COVID-19. In addition, this subgroup had significantly more young people who were under the adult age and could not be vaccinated because of the absence of development for this age vaccine in Russia at the time of the study. The absence of foreign vaccines in Russia is also important since every fifth respondent in this subgroup would like to be vaccinated with a foreign-made vaccine. Many students live with their families in Baku, and additional motivation for them to be vaccinated may concern elderly relatives and children. It should be noted that among Russian first-year students, a greater number of individuals have a negative attitude toward SARS-COV-2 vaccination and consider the rapidly developed SARS-COV-2 vaccines to be ineffective.

The percentage of medical students willing to recommend COVID-19 vaccination and thereby contribute increasing the collective immunity level is significantly dependent on the percentage of students who have been COVID-19 vaccinated [21]. So it is very important to achieve high rates of COVID-19 vaccination coverage in this group as they will counsel vaccine-hesitant people [26-28]. Medical students from Moscow and Baku branches indicated that the main reason for COVID-19 vaccination hesitancy among medical students was the lack of reliable information about SARS-COV-2 vaccines, which was also emphasized by other scientists [21, 26, 29, 30]. It was proved that the percentage of students who decided to be vaccinated against SARS-CoV-2 was positively correlated with the percentage of students whose main sources of information were medical professionals, medical universities, scientific conferences, and manuscripts that currently provide the least information about active SARS-CoV-2 immunization to medical students [21]. Public attitudes toward vaccination are formed by communication with medical students and directly depend on their competence in this issue, which was also noted by other authors [31, 32]. The ability to competently convey the immunoprophylaxis essence to the patients, to justify its necessity, to explain the existing questions in an accessible language, to inform about possible adverse reactions are the most important student skills they should receive, first of all, in a medical university [23].

For the first time, it was found that all vaccinated students, regardless of their place of study, were characterized by significantly greater psychological stability under conditions of rapidly increasing COVID-19 incidence during the fourth wave of the SARS-COV-2 pandemic, which is especially important for educational process efficiency and the further professional career of future specialists [15, 16].

One of our study limitations was the lack of result separations by sex. In addition, the relatively small number of males compared to females may likely limit the study results. In further studies, we will choose a proportional number of students. Another limitation is that the survey was conducted only among. That is why this study and results must be read as an initial stage of multi-central research on the COVID-19 vaccination impact on the psychoemotional student state.

## CONCLUSION

Thus, vaccination coverage of first-year students of Sechenov University during the fourth wave of the SARS-COV-2 pandemic was insufficient: 53.53±7.78%. At the Moscow branch, it was shown to be 42.9±7.69%; at the Baku branch − 69.6±7.69%. The lack of reliable information about SARS-COV-2 vaccines, their composition, action mechanisms and contraindications, indicated by a third of all respondents, may largely determine the motivated participation of first-year medical students in the vaccination SARS-COV-2 campaign. The role of a medical university in imparting knowledge about active SARS-COV-2 immunization to medical students was found to be insignificant. It was established that vaccination is a significant factor in stabilizing the psycho-emotional state of first-year medical students, which significantly affects the quality of their educational process and its effectiveness.

The obtained results support the following recommendations: 1. Vaccination is a compulsory event during a pandemic since it is not only a good preventive measure against the infection spread but also a significant factor in stabilizing the psycho-emotional state of medical students that affects the quality of their educational process. 2. The primary task is eliminating the information deficit about vaccines among medical students using professional educational resources from a medical university. 3. Introducing a vaccine for juveniles and registering foreign SARS-COV-2 vaccines in Russia will increase the percentage of vaccination among students and the population. 4. The informed decision of medical students to participate in the SARS-COV-2 vaccination campaign will contribute to their competent education of the population about SARS-COV-2 immunoprophylaxis and increase collective immunity in Russia.

## Figures and Tables

**Table 1:** Number, sex-age structure, and vaccination coverage of the first-year medical students.

-	First Moscow State Medical University named after I.M.Sechenov (Sechenov University)		
-	Moscow Branch, Russia	Baku Branch, Azerbaijan	Total
Respondent number	357 (60.1%)	237 (39.9%)	594 (100%)
Girls Youths	285 (79.8%) 72 (20.2%)	174 (73.4%) 63 (26.6%)	459 (77.3%) 135 (22.7%
Average age	18.10±1,78	17.89±0,48	17.89±0.61

**Table 2:** The epidemiological situation for SARS-COV-2 during the study.

Epidemiological Indicators	Moscow, Russia	Baku, Azerbaijan
Total incidence per 1000 people	144,17	52,53
Total mortality per 1000 people	2,48	0,70
Lethality, %	1,72	1,31
Vaccination coverage of the population, %	48,60	46,26

## Data Availability

All the data and supportive information are provided within the article.

## LIST OF ABBREVIATIONS

COVID-19: = Corona Virus 19
VECTOR: = Visualisation of the Exposure of Cyclists to Traffic on Roads
