# Supplementary material for: Vaccination as a Significant Factor Influencing the Psychoemotional State of Medical Students During the Sars-Cov-2 Pandemic: An International Aspect
Source: Clin Pract Epidemiol Ment Health. 2023 Jul 11;19:e174501792304060. doi: 10.2174/1745-0179-v19-e230420-2022-49 (PMC10487339; doi:10.2174/1745-0179-v19-e230420-2022-49)
Supplement: Supplementary file 1 — Supplementary material is available on the Publisher’s website. [file CPEMH-19-E174501792304060_SD1.pdf]

1745-0179/23

Send Orders for Reprints to reprints@benthamscience.net

Clinical Practice & Epidemiology in Mental Health

Content list available at: https://clinical-practice-and-epidemiology-in-mental-health.com

Supplementary Material

Vaccination as a Significant Factor Influencing the Psychoemotional State of Medical Students During the Sars-Cov-2 Pandemic: An International Aspect

Maria V. Sankova[1], Vladimir N. Nikolenko[1,2], Tatiana M. Litvinova[1], Beatrice A. Volel[1], Marina V. Oganesyan[1], Andjela D. Vovkogon[1], Negoria A. Rizaeva[1], Sergey V. Sankov[1] and Mikhail Y. Sinelnikov[1,3,*]

[1]*First Moscow State Medical University named after I.M.Sechenov, Sechenov University, 119991, Russia, Moscow, st. Trubetskaya, 8, bld. 2*
[2]*Lomonosov Moscow State University, 119991, Russia, Moscow, Leninskie Gory, 1*
[3]*Research Institute of Human Morphology, Moscow, Russian Federation*

Personal Information

Region

Moscow

Baku

Faculty

Medical

Pediatric

Dental

Pharmacy

Preventive-medicine

Academic year

1-3

4-6

Gender

Male

Female

Age

Your residence conditions

In a family

Alone or in university dormitory

Part 1 - Please answer the following questions truthfully. Be as thorough as possible:

1. Do you consider Sars-Cov-2 to be a dangerous disease ?

Yes

No

2. Were there cases of COVID-19 among your relatives?

Yes

No

3. Was there a fatal outcome among relatives or acquaintances because of COVID-19?

Yes

No

4. Were you sick of COVID-19? How hard ? Please answer the questions using a scale from 1-10, where 0 is 'wasn't sick at all' and 10 is 'was extremely sick'

0 1 2 3 4 5 6 7 8 9 10

5. Are you following the anti-epidemic recommendations, such as wearing masks in public places, hand sanitizing and social distancing?

Yes

No

6. Rate the effectiveness of non-specific SARS-CoV-2 preventive measures from 0 (completely ineffective) to 10 (very effective).

0 1 2 3 4 5 6 7 8 9 10

Part 2 - Please answer the following questions truthfully. Be as thorough as possible:

1. Do you think it is reasonable to get COVID-19 vaccinated during a pandemic?

Yes

No

2. What is your attitude towards COVID-19 vaccinaton?

Positive

Negative

I'm not sure about this question

3. What in your opinion gives COVID-19 vaccination?

Prevents **COVID-19** disease

Prevents **COVID-19** complications and severe disease forms

Nothing

4. Who needs to be COVID-19 vaccinated ?

Risk groups

Adult population

Kids

Adults and kids

Nobody

Part 3 - Please answer the following questions truthfully. Be as thorough as possible:

1. Do you think what COVID-19 vaccines are better?

Foreign

Russian

All vaccines are effective

All vaccines are ineffective

2. What COVID-19 Russian vaccine do you think is safety and effective?

"Gam-COVID-Vac" ("Sputnik V")

"EpiVacCorona" and "EpiVacCorona H"

CoviVak

Sputnik-Light

3. Are you sufficiently informed about the COVID-19 vaccination purpose, COVID-19 vaccine composition, their action mechanisms, their benefits and side effects ?

Yes, enough

No, not enough

4. What are your main sources of information about COVID-19 vaccines?

Internet

Medical workers

Scientific conferences and articles

University lecturers

Mass media

Familiar

I'm not interested in this issue

5. Are you satisfied with the received information about COVID-19 vaccines?

Yes

No

6. Do you recommend COVID-19 vaccination to your friends and relatives?

Yes

No

7. What should be the COVID-19 vaccination during a pandemic ?

Voluntary

Obligatory

8. Rate the COVID-19 vaccination effectiveness from 0 (completely ineffective) to 10 (very effective).

0 1 2 3 4 5 6 7 8 9 10

Part 4 - Please answer the following questions truthfully. Be as thorough as possible:

Have you been vaccinated against COVID-19?

I Yes

II No

**I If you have been vaccinated**	**II. If you haven't been vaccinated**
**Have you had side effects because of COVID-19 vaccination?**	**Reasons for not vaccinating**
No	Own negative experience of previous vaccinations
General weakness	Medical contraindications
Pain at the injection site	The presence of antibodies against Sars-Cov-2
Rise in temperature	Lack of reliable information about COVID-19 vaccines
Headache	Fear of side effects and post-vaccination complications
Muscle pain	Doubts about the effectiveness and safety of COVID-19 vaccines
Other	Negative attitude towards COVID-19 vaccination
**Why did you decide to get vaccinated?**	Opinion that it is better to get over Sars-Cov-2
To get a vaccination certificate	Lack of time and desire
For the COVID-19 prevention and it's severe forms	Lack of imported COVID-19 vaccines
For the COVID-19 prevention in cohabiting relatives	

© 2023 The Author(s). Published by Bentham Science Publisher.

This is an open access article distributed under the terms of the Creative Commons Attribution 4.0 International Public License (CC-BY 4.0), a copy of which is available at: https://creativecommons.org/licenses/by/4.0/legalcode. This license permits unrestricted use, distribution, and reproduction in any medium, provided the original author and source are credited.
